# Characterization of Starches Isolated from Colombian Native Potatoes and Their Application as Novel Edible Coatings for Wild Andean Blueberries (*Vaccinium meridionale* Swartz)

**DOI:** 10.3390/polym11121937

**Published:** 2019-11-25

**Authors:** Carolina Medina-Jaramillo, Santiago Estevez-Areco, Silvia Goyanes, Alex López-Córdoba

**Affiliations:** 1 Facultad Seccional Duitama, Escuela de Administración de Empresas Agropecuarias, Universidad Pedagógica y Tecnológica de Colombia, Carrera18 con Calle 22 Duitama 150461, Boyacá, Colombia; caromedina1986@gmail.com; 2 Facultad de Ciencias Exactas y Naturales, Departamento de Física, Laboratorio de Polímeros y Materiales Compuestos (LP&MC), Instituto de Física de Buenos Aires (IFIBA-CONICET), Universidad de Buenos Aires, Buenos Aires C1428, Argentina; sestevezareco@df.uba.ar

**Keywords:** native potatoes, Andean blueberry, edible coatings, post-harvest, Starch, biopolymers

## Abstract

Andean blueberry is a promissory fruit native to South America. The current work aimed to characterize starches isolated from Colombian native potatoes and to evaluate the effect of the application of starch edible coatings on the changes in the physicochemical quality parameters of the Andean blueberry during storage. Starches were isolated from three different potatoes varieties (pacha negra, mora, and alcarrosa) and characterized. Then, starch-based coatings were applied to Andean blueberries, and the changes in their quality parameters were monitored during 12 days of storage. Despite the phenotypical differences in the starch sources used, starches were similar in terms of their granule morphology, amylose content (~19%), crystallinity degree (~46%), and thermal properties. Coatings were able to reduce the gaseous exchange of the fruit, and, thus, the respiration rate of all coated blueberries was ~27% lower compared to the uncoated fruits (*p* < 0.05) at the end of the storage. While the application of starch coatings did not prevent water loss, all samples reached water loss of up 20%. Besides, the coated fruits showed soluble solids contents ~14% higher compared to the control one, as well as better bright and firmness. The new edible coatings can help add value to the Andean blueberry.

## 1. Introduction

The use of naturally renewable biopolymers (e.g., starch, cellulose, chitosan/chitin, caseinate, and pectins) as edible and biodegradable films and coatings constitute an actual and sustainable choice to prevent the post-harvest losses of very perishable fruits, such as blueberries.

The production of blueberries (family Ericaceae, genus *Vaccinium*) has been rapidly growing in the last years, reaching over 596,813 t worldwide in 2017, being led by the United States with 40% of total production and Canada with 27% [1]. Highbush blueberries (*Vaccinium corymbosum*) are the most commercial blueberries. However, several other wild shrubs of the genus *Vaccinium* also produce commonly eaten blueberries, such as the European *Vaccinium myrtillus*.

*Vaccinium meridionale* Swartz (Andean blueberry) is one of the species of the genus *Vaccinium* that grows in the Andean region of South America at 2300–3300 m above sea level (m.a.s.l.) [2]. The fruit is a berry, 5–10 mm in diameter, dark reddish, with a sour and tart character. In Colombia, Andean blueberry is sold at the local markets, and it is eaten fresh, dried, and cooked into sauces, jellies, and jams [3]. *Vaccinium meridionale* Swartz is considered to be health-promoting food due to its high content of anthocyanins (~329 ± 28 mg/100 g, mainly cyanidin 3-glucoside), and phenolic acids (~ 758.6 ± 62.3 mg gallic acid equivalent/100 g, mainly chlorogenic acid) [3]. Several studies have shown than Andean blueberry has antioxidant, anticarcinogenic, and anti-inflammatory properties [3,4,5]. Therefore, *Vaccinium meridionale* Swartz has a high potential of use as an antioxidant additive or functional ingredient in food, pharmaceutics, and cosmetic applications [6].

As for most blueberries fruit, Andean blueberries are perishable fruits, and, therefore, it is necessary to develop strategies to increase their storage life [7]. It has been reported that the fruit decays quickly at ambient temperature after harvest, thus affecting its commercialization [8]. Moreover, blueberry fruits are susceptible to bruising from mechanical impact, water loss, and microbial attack [9,10].

Coatings are defined as mixtures of film-forming materials plus solvents and other additives (e.g., plasticizers), which, when applied to a surface and cured or dried, yield a solid protective, decorative and/or functional adherent thin layer [11]. Moreover, coatings can be added of antioxidants, antimicrobial agents, oxygen scavengers, or moisture absorbing, among others, to obtain active coatings [12,13]. Over the past years, the use of coatings has become more and more important in the food field. It has been reported that the application of coatings allows extending the shelf life of perishables and sensitive products such as fruits and vegetables because these materials act as an external protective layer slowing the respiration rate and reducing moisture and solute migration, gas exchange, oxidative reaction rates, as well as suppress physiological disorders of fresh-cut fruits [12,14,15]. Several polymers from natural origin have been used for the fabrication of food edible coatings, including starch [16], alginate [17], chitosan [18], pectin [17] and cellulose derivatives [19], among others. Between them, starches are well known for their good film-forming properties and functionalities [20]. Moreover, it has been stated that because the edible films and coatings based on starches are reported to be transparent, odorless, tasteless, and colorless, they would not exhibit adverse effects on the sensory quality of a product [21,22,23].

Despite that starches from diverse sources have been widely used as edible coating material and there is a tremendous amount of knowledge on composition, structure, properties, and modifications [24,25,26], starches from Colombian native potatoes used in this research have not been studied until now. It is commonly hypothesized that starch granules architecture varies widely between different botanical sources, and so has a significant impact on its physicochemical properties [24,25,26]

In the current work, starches were isolated from three different Colombian potatoes varieties (mora, pacha negra, and alcarrosa), and their physicochemical properties were investigated and compared. Edible coatings based on the different starches were applied on Andean blueberries, and the changes in fruit physicochemical parameters were monitored during 12 days of storage. As far as the authors are aware, this is the first time that starches isolated from Colombia native potatoes have been characterized in detail and applied as edible coatings on Andean blueberry.

## 2. Materials and Methods 

### 2.1. Materials

**Figure 1:**
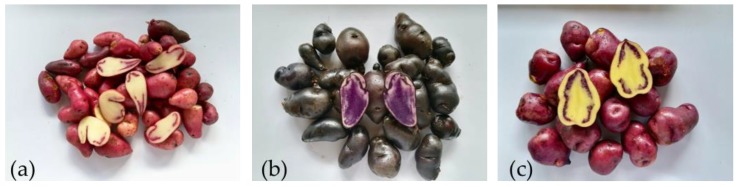
Colombian native potatoes used in this study: (**a**) pacha negra, (**b**) mora, and (**c**) alcarrosa.

Starches were isolated from three varieties of native potatoes (*Solanum tuberosum*), grown in Ventaquemada (Boyacá, Colombia) at 2630 (m.a.s.l.) (Figure 1).

Andean berries (*Vaccinium meridionale* Swartz) at maturity stage 4 (100% purple) and dry content (%) of 23.58 ± 1.90 were obtained from a local supermarket (Duitama, Colombia). The berries were examined previous to its use to separate fruits with physical, mechanical, or microbial damages. All chemicals used were of analytical grade. Dimethyl Sulfoxide was purchased from Sintorgan^®^ (Buenos Aires, Argentina). Urea was purchased from YPF (Buenos Aires, Argentina). Iodine and Potassium iodide were purchased from Anedra (Buenos Aires, Argentina). Ethanol 95.5% was purchased from Merck^®^ (Darmstadt, Germany). Glycerol was purchased from J. T. Baker (Phillipsburg, NJ, USA). Sodium hydroxide was purchased from Sigma Aldrich (St. Louis, MO, USA).

### 2.2. Isolation and Characterization of Starches

#### 2.2.1. Starches Isolation and Yield

Starches isolation was carried out from each potato cultivar according to the optimized protocol by Doporto, Dini, Mugridge, Viña, & García, 2012 [27]. Briefly, potatoes were washed, sanitized (250 ppm of chlorine, 10 min), peeled and pulped with a grater. The grated potatoes were blended with water (2 L water/kg) and stored at 4 °C for 24 h. The blend was filtered using a cheesecloth, and the starch slurry was decanted at 4 °C. The supernatant was discarded, and the starch cake was recovered, dried at 40 °C for 14 h in a hot-air oven, and milled.

The starch yield was calculated as the percentage of dry weight (dehydrated at 40 °C) of isolated starch related to the fresh weight of the potato tuber.

#### 2.2.2. Amylose Content 

Amylose content of starches was determined by the iodine binding method [28]. An amount of each potato starch was weighted (*m_starch_*) and mixed with 10 mL of urea-DMSO solution (1:9 v/v). The blend was heated in an oven at 100 °C for 1 h and cooled to room temperature. Then, 0.5 mL of the prepared solution were weighed (*m_solution_*) and diluted in 5 mL of ethanol. The solution was centrifuged (5000 rpm, 30 min) and the supernatant was discarded. Urea-DMSO solution (1 mL), I_2_/IK solution (1 mL) and distilled water (to a final volume of 50 mL) were incorporated. Absorbance was measured at 635 nm, and amylose content was calculated according to the following equation:

(1) $$Amylose\;content\;\left(\%\right)=28.414\times \frac{A_{635}}{2\times m_{starch}\times m_{solution}}\times 100$$

where *m_starch_* is the amount of each potato starch used in the assay, *m_solution_* is the weight of the solution, and *A*_635_ is the absorbance measured at 635 nm.

#### 2.2.3. Size and Morphology of Starch Granules 

The morphology of potato starch granules was examined by scanning electron microscopy using a field emission gun (FE-SEM) (SUPRA 40, Carl Zeiss NTS, Oberkochen, Germany). Starch granules were attached to aluminum stubs using a two-sided adhesive tape and sputtered with a thin layer of platinum before observation.

Size distributions of granules were determined using a Laser Diffraction Particle Size Analyzer SALD-3101 (LS, Shimadzu, Kyoto, Japan). For this purpose, starches were dispersed in ethanol (96% *v/v*) and diluted until an absorbance between 0.12 and 0.20 was obtained.

#### 2.2.4. Infrared Spectra

FTIR analysis was performed in a Jasco FT-IR 4100 spectrometer (Hachioji, Tokyo, Japan) equipped with attenuated total reflectance (ATR) module. Samples were placed on the ATR accessory, and they were analyzed, taking 64 scans per experiment with a resolution of 4 cm^−1^.

#### 2.2.5. X-ray Diffraction Patterns 

X-ray diffractograms of the starch granules were recorded at 2θ between 5° and 50° with a step size of 0.02° in a Philips-APD PW 1710 diffractometer (Eindhoven, North Brabant, The Netherlands) using Cu-Kα radiation (λ = 1.542 A). The crystalline fractions were calculated as reported Lopez-Rubio, Flanagan, Gilbert, & Gidley [29]. Patterns were fitted with an amorphous background and several crystalline peaks using Gaussian functions. The fitting coefficients were refined to minimize the value of Chi-squared using the Levenberg-Marquardt algorithm. The crystalline fraction was calculated according to Equation (2):

(2) $$X_{c}=\frac{\sum^{\;}Ac_{i}}{A_{t}}$$

where $X_{c}$ corresponds to the crystalline fraction, $Ac_{i}$ to the area of each crystalline peak, and $A_{t}$ to the total area. The fitting procedure was repeated five times with different initial inputs.

#### 2.2.6. Thermal Properties 

The temperature and the enthalpy of gelatinization of the starch granules were determined by differential scanning calorimetry (DSC) (Mettler Toledo, Schwerzenbach, Switzerland). The starch samples (~2.5 mg) were weighed into aluminum DSC pans, to which 7.5 mg of deionized water was added. After sealing, the pans were heated from room temperature to 90 °C with at a heating rate of 10 °C /min, under nitrogen atmosphere (30 mL/min). An empty aluminum pan was used as a reference. The onset (*T*_on_), peak (*T*_p_), conclusion (*T*_c_) temperatures, and gelatinization enthalpies (Δ*H*) were estimated using the TA Instrument universal analysis software.

Simultaneous Thermogravimetric/Differential thermal analyses (TGA/DTA) were performed in a Shimadzu DTG-60 (Kyoto, Japan). Dry samples (~5 mg) were placed in aluminum pans inside the thermogravimetric balance and then heated under a nitrogen atmosphere (20 mL/min) in a range of 30 to 450 °C at a heating rate of 10 °C/min. TGA curves were derived with respect to temperature to determine the degradation temperatures.

### 2.3. Preparation and Application of Starch Edible Coatings

The preparation of edible starch coatings was carried out as reported in a previous work [30]. Blends of each potato starch (2.0 g), glycerol (0.6 g) and distilled water (97.4 g) were homogenized for 40 min and then heated to 92 °C, under constant stirring, until complete starch gelatinization. The formulations were cooled to room temperature for later application on the fruits.

A total mass of 6 kg of blueberries was randomly divided into four groups (control, pacha negra starch edible coating, mora starch edible coating, alcarrosa starch edible coating); each group containing 1.5 kg of Andean blueberries. The fruits were dip-coated by immersion in the coating solutions for 90 s, drained of excess coating, and air-dried at room temperature. Control samples (without coating) were also prepared by immersing of fruits in distilled water and kept under the same storage conditions than treated ones, for comparison.

### 2.4. Evaluation of Quality Attributes of Andean Blueberries along Storage 

Coated and uncoated Andean blueberries were packed in PET trays with perforated vents and stored for 12 days. Evaluations of quality attributes were performed at 1, 5, 9, and 12 days of storage at room conditions (14 °C and 80% RH). For every sampling time, three trays containing 125 g (~250 units) of Andean blueberries each were prepared.

#### 2.4.1. Respiration Rate

Respiration rate was measured as reported Hasperué, Rodoni, Guardianelli, Chaves, & Martínez, 2016 [31]. Approximately 120 g of Andean blueberries were placed for 30 min at 25 °C inside hermetically sealed 2 L flasks. Then, the CO_2_ concentration was determined using an infrared analyzer (LabQuest^®^2 Model LQ2-LE, Beaverton, OR, USA), and the results were expressed as the rate of respiration (CO_2_) in mg kg^−1^ s^−1^.

#### 2.4.2. Soluble Solids Content, pH and Titratable Acidity (%)

The soluble solids content was measured in the fruit juice using an Atago refractometer model PR 101 (Atago CO., Tokyo, Japan) and expressed as °Brix (AOAC 932.12). Fruit samples were crushed using a blender and filtered through filter paper to obtain the fruit juice. The pH of the fruit samples was assessed using a digital pH meter (Oakton Instruments, Vernon Hills, IL, USA) (AOAC 981.12).

Titratable acidity (%) was determined by titration with 0.1 N NaOH up to pH 8.1, using 1 g of sample in 10 mL of distilled water (AOAC 942.15). The results were expressed in citric acid percentage.

#### 2.4.3. Weight Loss 

Weight loss of Andean blueberries during storage was determined by weighting all fruit trays at the beginning of the storage and every day of analysis. The weight loss (% *W*) was calculated with the following equation:

(3) $$\%\;W=\;\left(\frac{m_{0}-m_{f}}{m_{0}}\right)x\;100$$

where *m_f_* is the weight at each time and *m*_0_ the initial weight of each sample

#### 2.4.4. Firmness Analysis

Firmness was determined using a digital Force Gauge PCE-FM200 (Southampton, UK) equipped with a 6 mm diameter stainless steel probe. Firmness was defined as the maximum force to disrupt the tissue at the penetration time used (5 s) [32]. The results were expressed as an average of at least five measurements.

### 2.5. Statistically Analysis

The statistical analysis was performed using the Systat Inc. software (Evanston, IL, USA). Analysis of variance (ANOVA) and Tukey pairwise comparisons were carried out using a level of 95% confidence. The experiments were performed at least in duplicate, and the data were reported as mean ± standard deviation.

## 3. Results and Discussion

### 3.1. Characterization of Starches 

Table 1 shows the characteristics of the starches isolated from the Colombian native potatoes. All three starches had similar isolation yield (~11%). It was recently reported isolation yields (%) ranging between 7.0% and 16.5% when worked with yellow skin potatoes for extraction of starch [33]. Concerning the amylose content (%), alcarrosa starches showed higher amylose content than the mora and pacha negra starches, even though these differences were not statistically significant (*p* > 0.05) (Table 1). Amylose/amylopectin ratio is a leading parameter since it determines the physicochemical properties of starches, such as gelatinization energy and crystalline structure [34]. Besides, the properties of starch coatings are also affected by the amylose/amylopectin ratio. Generally, starch films with higher amylose content present a better barrier to oxygen [35]. According to the literature, the amylose content of potato starch generally varies between 18% and 30% [25,34], indicating that the potato varieties used in this work are of relative low amylose content.

**Figure 2:**
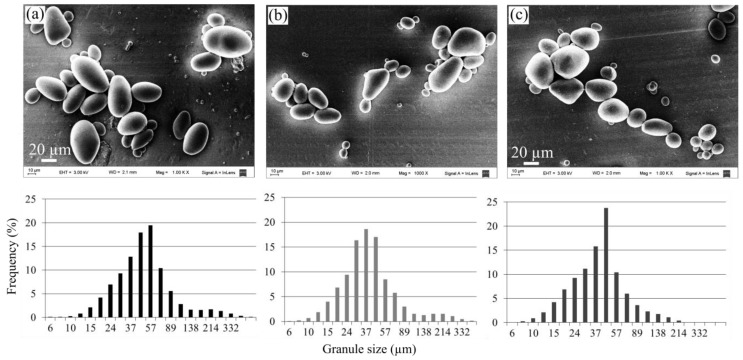
SEM images and size distributions of the starch granules: (**a**) pacha negra starch, (**b**) mora starch, and (**c**) alcarrosa starch.

Size and morphology of starch granules depend strongly on the botanical source. Besides, these parameters affect the properties of the corresponding starch gel directly; therefore, the different isolated starches were characterized by means of their granule sizes and morphology. Figure 2 presents SEM images and size distributions of the different samples, and the mean diameters of the granules are reported in Table 1. All the starches showed an average granule size of ~47 µm without significant differences between them. Besides, a wide distribution of granule size (Figure 2) was observed in all systems. Starch granules resulted mostly ellipsoidal, but some small granules were circled shaped, and some large granules were irregularly shaped. Size and morphology of granules resulted similarly to those reported in the literature for potato starches from different varieties [36,37,38].

**Figure 3:**
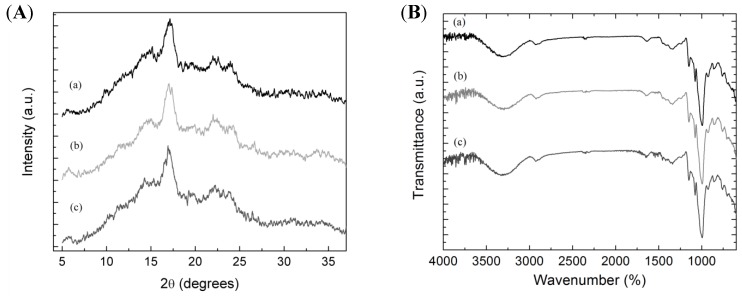
XRD diffraction patterns (**A**) and FTIR spectra (**B**) of the different starches isolated from the Colombian native potatoes studied: (**a**) pacha negra starch, (**b**) mora starch, and (**c**) alcarrosa starch.

Figure 3a shows the X-ray diffraction patterns of the studied samples, and the percentages of crystallinity are reported in Table 1. Similar crystalline fractions were obtained for all the studied samples (~45%), and the obtained values were in agreement with the literature (15–45%) [39,40]. Starches showed a semi-crystalline structure with diffraction peaks centered at 5.7°, 14.7°, 17.1°, 19.7°, 22.1°, 24.0°, 26.6°, 30.6°, and 34.7°. These patterns correspond to a B-type structure, which is characteristic of potato starch [41] and consists of double helices of amylose packed with a P61 space group in a hexagonal unit cell [42]. Some differences could be observed in the obtained diffraction patterns. The intensity of the crystalline peaks centered at 5.7° and 26.6° was slightly higher in mora starch, while the intensity of the peaks at 14.7° and 19.7° was slightly higher in alcarrosa starch. Besides, alcarrosa starch showed an additional peak at 23.1°. These differences indicate that polymer packing is different, which could be attributed to different molecular weight distributions of amylose and amylopectin in each potato starch variety.

FTIR constitutes a standard method to study chemical interactions in polymers and soft matter. Mainly it is useful to characterize starch granules concerning its short-range structure. FTIR spectrum of the studied systems showed the characteristic peaks of starch: O–H stretching (3300 cm^−1^), C–H symmetric and asymmetric stretching (2930 and 2890 cm^−1^), C–O stretching (1149 cm^−1^), C–C stretching (1077 cm^−1^), and C–OH bending (1100–900 cm^−1^) (Figure 3b) [43]. No additional bands were observed in any sample, which suggests that the isolation procedure was effective. Besides, no significant differences were observed between the spectra of the different potatoes starches, indicating that interactions between polymer chains are similar for all systems. In particular, the bands in the region 1100–900 cm^−1^ are highly sensitive to changes in the short-range structure. Since there were no differences in the relative intensity of the bands in this region, it can be concluded that the short-range structure is similar for all the studied starch granules.

**Figure 4:**
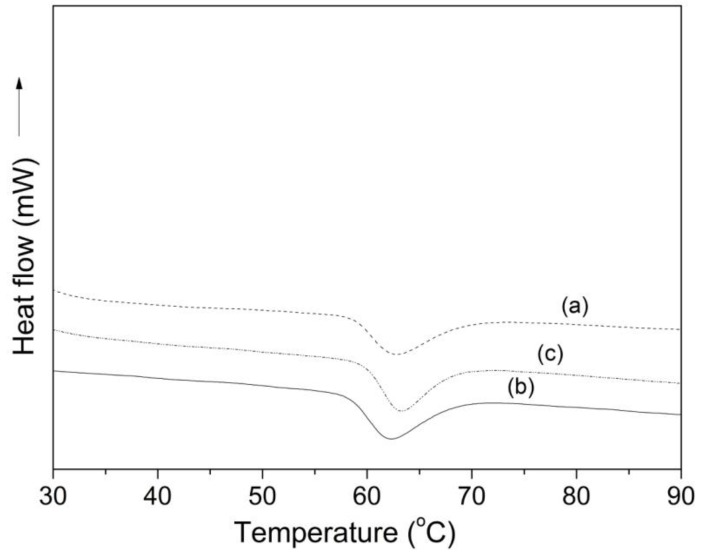
DSC thermograms of starches isolated from the different Colombian native potatoes: (**a**) pacha negra, (**b**) mora and (**c**) alcarrosa.

Figure 4 shows the DSC thermograms of the starches isolated from the different Colombian potato cultivars. All samples showed similar temperatures (*T*_on_ ~ 58 °C; *T*_p_ ~ 62.8 °C; *T*_c_ ~ 70.1 °C) and enthalpies (∆*H* ~ 12 J/g) of gelatinization. Martínez et al., 2019 reported similar thermal gelatinization properties when worked with starches isolated from native potatoes of the Andean region (Imilla blanca, Imilla negra and, Loc’ka).

**Figure 5:**
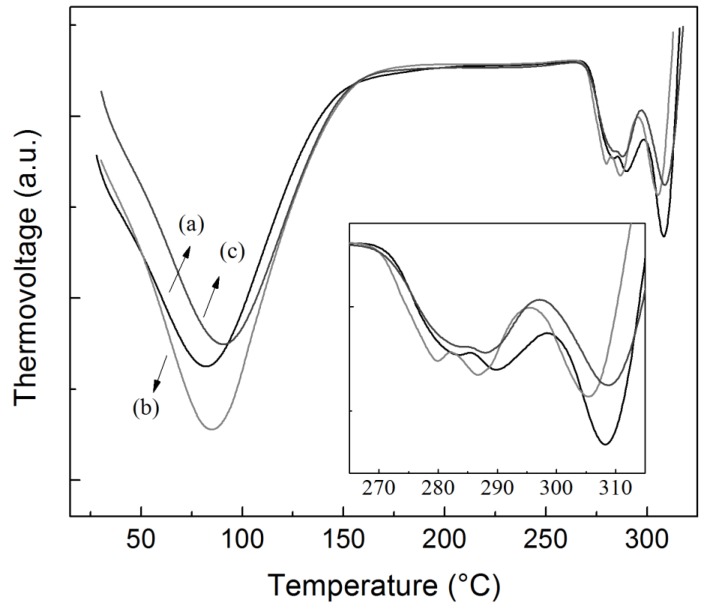
Differential thermal analyses (DTA) curves of the different starches isolated from the Colombian native potatoes studied: (**a**) pacha negra starch, (**b**) mora starch, and (**c**) alcarrosa starch.

On the other hand, the thermal degradation of all samples occurred in two steps. The first event occurred between 50 and 150 °C and corresponds to moisture loss, while the second event occurred between 250 and 342 °C, with the maximum of the peak at 305 °C, and corresponds to thermal decomposition of the starch polymer [44]. No significant differences were detected between the samples (TGA curves not shown). Figure 5 presents the DTA curves of the different starch granules. In all samples, four endothermic processes were observed. The first event occurred between 30 and 160 °C and corresponded to the melting of crystallites [45]. The rest of the thermal events occurred between 270 and 320 °C and were associated with the thermal degradation of starch (according to TGA results). Generally, the thermal degradation of starch reveals two endothermic peaks corresponding to the degradation of amylose (T ~ 275 °C) and amylopectin (T > 300 °C) [46]. However, in this work, the degradation of starch consisted of three endothermic peaks (insert in Figure 5). The thermal event at higher temperatures was associated with amylopectin degradation, and it was centered at 308 °C for pacha negra and alcarrosa starches, and at 305 °C for mora starch. Then, the peaks at lower temperatures were associated with the degradation of amylose fractions with different molecular weights. Tong et al. [25] recently characterized different pigmented potatoes from China and reported a binomial distribution of amylopectin molecular weight. The endothermic peaks observed in this work were centered at 283 °C and 290 °C for pacha negra starch, at 279 °C and 286 °C for mora starch, and at 283 °C and 288 °C for alcarrosa starch. Both amylose and amylopectin degradation temperatures were found at lower temperatures for the samples corresponding to mora starch.

### 3.2. Effect of Edible Coating on the Visual Appearance of Andean Blueberry

**Figure 6:**
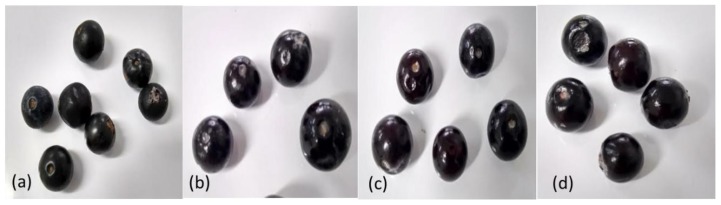
Images of Andean blueberries without and with coatings: (**a**) control; (**b**) pacha negra starch coating; (**c**) mora starch coating, and (**d**) alcarrosa starch coating.

Figure 6 shows images of Andean blueberries without and with edible starch coatings. It can be seen that the coatings-forming solutions form a good film on the surface of the Andean blueberries, giving the fruit a brighter, translucent, fresh-like appearance, compared to the control fruit. It has been reported that edible coatings have useful effects on the bright appearance of the fruits due to that the coating forms a smoother surface compared to the fruit skin. In this sense, a surface with less roughness would lead to a greater reflection of visible light [47].

### 3.3. Changes in Andean Blueberry Quality Parameters during Storage

**Figure 7:**
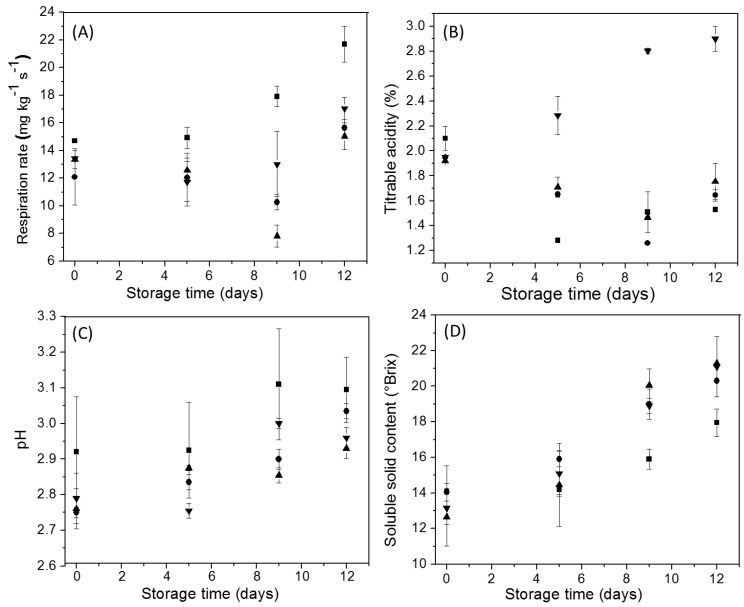
Changes in Andean blueberry quality parameters during storage: Respiration rate (**A**), Titratable acidity (**B**), pH (**C**), and Soluble solids content (**D**). Control (■), pacha negra starch edible coating (▲), mora starch edible coating (●), alcarrosa starch edible coating (▼).

Figure 7A shows the changes in the respiration rate (%) of uncoated and coated Andean blueberries along storage. In general, the respiration rate of the fruits was decreased by the application of the edible coatings based on all potato starches studied. At the end of the storage, the respiration rate of the coated Andean blueberries was ~27% lower compared to the uncoated fruits (*p* < 0.05), regardless of the starch type used. It has been reported that polysaccharide coatings exhibit a high barrier to oxygen, significantly limiting respiration of the fruit [48,49,50]. Control fruits showed a significant increase in their respiration rate after 5 days of storage (*p* < 0.05); while in the samples with edible coatings based on mora and alcarrosa starches, significant increases were not found along the assay (*p* < 0.05). The samples coated with pacha negra starch showed a significant descent in the respiration rate at 9 days of storage and then an increase at the end of storage. This behavior was probably due to that with the passing of the days of storage, the oxygen permeability of the pacha negra edible coatings may have been improved. However, at the end of storage, the water loss of the fruit can be slightly affected the integrity of the edible coating, and therefore their efficiency facing the gaseous exchange was decreased.

For what concern the titratable acidity (%), uncoated Andean blueberries decreased their acidity (%) as from 5 days of storage, in relation to their initial value (Figure 7B). Similar behavior was observed for the samples with edible coatings based on mora and pacha negra starches (Figure 7B). A decrease in the titratable acidity (%) of Andean blueberries stored at different temperatures was reported recently [8]. It has been well documented that organic acids are primary substrates involved in the respiration process of climacteric fruits; thus, a reduction in acidity is commonly expected during fruit ripening [51,52]. Unlike the other starches, the edible coatings based on alcarrosa starch produced a significant increase in the titratable acidity (%) of Andean blueberries as from 5 days of storage (Figure 7B). This behavior was probably due to that the alcarrosa starch coatings could have acted delaying the utilization of organic acids as respiration substrate and providing higher rigidity to the skin of the fruit preventing the loss of the organic acids (e.g., citric acid). Similar observations have been reported by other authors [53,54]. The differences in the effect of alcarrosa starch edible coating on the changes in the titratable acidy during storage can also be associated with the slight differences in the amylose content of the starches (Table 1).

Concerning the pH, no relevant differences were detected among the uncoated and the differently coated samples at each considered storage time (Figure 7C).

**Figure 8:**
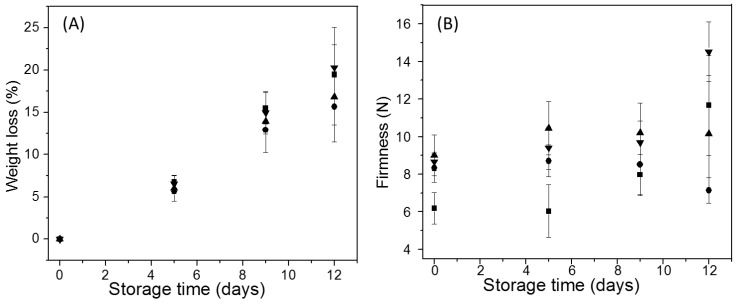
Behavior of the weight loss (**A**) and the firmness (**B**) of Andean blueberries during storage. Control (■), pacha negra starch coating (▲), mora starch coating (●) and, alcarrosa starch coating (▼).

On the other hand, Andean blueberries with and without edible starch coatings showed a gradual increase in their soluble solids content during storage (Figure 7D). After 5 days of the assay, statistically significant differences were found between the samples with and without starch coatings (*p* < 0.05). At the end of the storage, the soluble solids content of the coated Andean blueberries was 14% higher compared to the uncoated fruits (*p* < 0.05), and no significant differences were found between the different starches used. Generally, the concentration of soluble solids is associated with dehydration or weight loss. A more significant weight loss corresponds to a higher concentration of soluble solids. Moreover, it has been stated that quick respiration rates, in turn, accelerated the synthesis and use of metabolites that result in increasing the soluble solid concentration [55]. However, in the present work, a non-conventional behavior was observed; the coated fruits showed lower respiration rates (%) and similar weight loss (%) than the uncoated fruits (Figure 7A and Figure 8A). Therefore, it was hypothesized that edible coatings based on all the starches studied were able to act as an effective barrier avoiding the loss of soluble solids.

Figure 8A shows the changes in the water loss of uncoated and coated Andean blueberries along storage. All fruits showed a progressive increase of this water loss (%) until reach values of around 20% at the end of the storage, regardless of the presence of edible coatings. The loss of weight in fresh blueberries is mainly due to the loss of water caused by transpiration and respiration processes, which is determined by the gradient of water vapor pressure between the fruit and the surrounding air [56]. The water loss was apparently higher for the uncoated samples than for the coated fruits; however, there were no significant differences between all samples (*p* < 0.05). It has been reported that water loss depends on several factors such as fruit surface area/volume, fruit integrity, storage conditions (relative humidity and temperature), among others [53,54,55,56,57]. Rincón Soledad et al. reported water loss (%) for the Andean blueberry of 37.3% after 30 days of storage at 20 °C. Moreover, they stated that water loss higher than the 10% provoked changes in the freshness of the fruit [8]. The use of edible coating has been reported as an alternative to control the water loss of fruits. However, it has been stated that edible coatings based on natural polymers such as starches and chitosan did not act as an effective barrier against the weight loss [56,57,58].

At the beginning of storage, the firmness of all coated Andean berries was 41% higher compared to the uncoated fruits (*p* < 0.05). With the passing of 5 days of storage, the increase in the firmness of the coated samples was higher (~58%), with respect to the control samples (*p* < 0.05) (Figure 8B). At 9 day of storage, all samples showed similar firmness (*p* < 0.05). Among all treatments, Andean berries coated with alcarrosa starches were more firm, and the fruits coated with mora starches showed lower firmness at the end of storage. Mannozzi et al. observed an increase in the firmness of blueberries immediately after coating with chitosan, and this was attributed to that the coating provides rigidity to the skin of the fruit [18]. In the uncoated fruits and the samples with alcarrosa potato starch coating, their firmness increased around 75% and 58%, respectively, at the end of storage. It has been stated that water loss leads to increased firmness during post-harvest storage [7,8,9,10,11,12,13,14,15,16,17,18,19,20,21,22,23,24,25,26,27,28,29,30,31,32,33,34,35,36,37,38,39,40,41,42,43,44,45,46,47,48,49,50,51,52,53,54,55,56,57,58,59]. Besides, mora and pacha negra potato starch coatings allow preserving the firmness of the blueberries with the passing of the storage days. It has been reported that the fruit firmness commonly decreased along storage due to the ethylene production, which encourages the synthesis of enzymes responsible for softening such as polygalacturonase [57]. Therefore, it can be stated that mora and pacha negra potato starch coatings were able to maintain the firmness of the Andean blueberries along storage. This behavior is positive from a commercialization point of view because firmness is one of the factors that most influence the purchase intent of the consumers, who prefer fresh-like fruits.

## 4. Conclusions

Colombian native potatoes constitute an important source of starches that can be potentially used in the development of several new food and non-food products, including edible coatings. The application of edible coatings based on starches isolated from Colombian native potatoes proved to be a useful and straightforward method to decrease the respiration rate and the loss of soluble solids of Andean blueberries during storage. Moreover, the coatings maintained the firmness of the fruits. However, the application of these edible coatings did not avoid the water loss of the fruits during storage, and, therefore, more studies are necessary to improve the water barrier properties of the coatings. The new edible coatings can be helpful for fruit farmers and agro-food enterprises interested in adding value to their products thought the improvement of the appearance and the increase of the shelf life.

## Figures and Tables

**Table 1:** Characteristics of the starches isolated from the Colombian native potatoes.

Sample	Extraction Yield (%)	Amylose Content (%)	Mean Granule Size (µm)	Shape Description	Crystallinity Fraction (%)
Pacha negra starch	11.9 ± 1.5 ^a^	17.92 ± 0.26 ^a^	49.3 ± 1.7 ^a^	Ellipsoid	48.0 ± 1.6 ^a^
Mora starch	10.1 ± 1.3 ^a^	18.27 ± 0.59 ^a^	47.6 ± 1.5 ^a^	Ellipsoid	44.7 ± 2.2 ^a^
Alcarrosa starch	11.0 ± 1.2 ^a^	19.58 ± 0.62 ^a^	45.9 ± 1.7 ^a^	Ellipsoid	45.9 ± 3.9 ^a^

Different letters within the same column indicate statistically significant differences (*p* < 0.05).
